# Hyperspectral imaging for chemicals identification: a human-inspired machine learning approach

**DOI:** 10.1038/s41598-022-22468-7

**Published:** 2022-10-20

**Authors:** Shai Kendler, Ziv Mano, Ran Aharoni, Raviv Raich, Barak Fishbain

**Affiliations:** 1 Department of Environmental, Water and Agricultural Engineering, Faculty of Civil and Environmental Engineering, Technion – Israel Institute of Technology, Haifa, Israel; 2 Kelley Engineering Center, School of Electrical Engineering and Computer Science, Oregon State University, Corvallis, USA; 3 Present Address: Environmental Physics Department, Israel Institute for Biological Research, 24 Lerer St., 74100 Ness Ziona, Israel; 4 Physics department, Nuclear Research Centre - Negev, Beer Sheva, Israel

**Keywords:** Computational science, Scientific data

## Abstract

Data analysis has increasingly relied on machine learning in recent years. Since machines implement mathematical algorithms without knowing the physical nature of the problem, they may be accurate but lack the flexibility to move across different domains. This manuscript presents a *machine-educating* approach where a machine is equipped with a physical model, universal building blocks, and an unlabeled dataset from which it derives its decision criteria. Here, the concept of machine education is deployed to identify thin layers of organic materials using hyperspectral imaging (HSI). The measured spectra formed a nonlinear mixture of the unknown background materials and the target material spectra. The machine was educated to resolve this nonlinear mixing and identify the spectral signature of the target materials. The inputs for educating and testing the machine were a nonlinear mixing model, the spectra of the pure target materials (which are problem invariant), and the unlabeled HSI data. The *educated machine* is accurate, and its generalization capabilities outperform classical machines. When using the educated machine, the number of falsely identified samples is ~ 100 times lower than the classical machine. The probability for detection with the educated machine is 96% compared to 90% with the classical machine.

## Introduction

Since the seminal work by Samuel^1^, who coined the term Machine Learning (ML), this field has impacted virtually all areas of science, including engineering^2,3^, the exact and social sciences^4–6^, and even art^7^. The ML process can be described in the following way: let ${\mathbb{C}}$ be the set of possible states for the observed system. Let *O(X)* be a set of observations, each described by a set of features *X*, which a-priori are associated with a specific state $\mathcalligra{c} \in {\mathbb{C}}$; i.e., their states are known. *O(X)* is then used to train a machine and to establish a set of rules for associating observation *O(X)* and a system's state $\mathcalligra{c}$. When these different system states are viewed as different classes, this set of rules is referred to as a classifier. Using this classifier, the machine can classify new observations according to their feature values^8^. The classification accuracy depends on the nature of the training set, since it must adequately account for diversity in the test data. If *X* is a vector of the physical properties of an object that were measured experimentally, $O\left( X \right)$ must be chosen in such a way that the measurement noise of *X* is represented adequately. Take, for example, a situation in which each feature measurement is associated with additive random noise, such that $x \in X$ is of the form $x = x + RN_{x}$, where RN_X_ is the typical random noise of feature *x*. Hence, training should use observations that have similar noise characteristics, and the resulting classifier applies to observations in the same domain; i.e., that have these same noise characteristics^9,10^. Let us now consider the same problem as described above, but with multiplicative rather than additive noise; i.e., $x = x \cdot RN_{x}$. In this case, if the classifier that was trained on observations with an additive noise model is used as is to classify observations with multiplicative noise, the classification accuracy is likely to decrease. In this case, a new set of classified observations needs to be obtained and a new classifier trained, or at least attempts should be made to transfer the existing classifier to the new domain using a smaller set of labeled observations in the new domain^11,12^.

Despite these shortcomings, ML continues to have enormous appeal since a machine excels where humans struggle. Machines can handle vast amounts of high-dimensional data. In fact, ML improves with growth in the size of the database. Computer hardware and algorithms are constantly evolving and can handle large amounts of data and complicated problems^13^. On the other hand, humans can adapt to new situations, retrieve relevant knowledge, and outperform machines if the data and dimensionality are relatively small^14–16^.

The lack of large datasets limits the applicability of ML and Deep Learning (DL) and has driven several studies that tried to overcome this shortcoming. Raissi et al.^17^ introduced the Physics-informed neural networks (PINN), in which physics laws are used to restrict the possible solutions during the training stage of a neural network (NN). Introducing such a regularization mechanism results in a robust NN with a relatively small training set. Manome et al.^18^ described a method to automatically adjust the learning rate by incorporating human cognitive biases into the training process. Such biases mimic the human use of causal relationships between events in the learning process. Adding these biases results in a more accurate classification even with a relatively small dataset and eliminates the need for parameter tuning. Fong et al.^19^ described a paradigm for *neutrally-weighted* machine learning, which took functional magnetic resonance imaging (fMRI) measurements of human brain activity from subjects viewing images and used these data as part of the training process of an object recognition algorithm to benefit from the capabilities of the human brain. After training, image classification did not require fMRI input. This approach improved the classification accuracy by 10–30% when using traditional machine vision features and by 3–5% when using convolutional neural network features. While these ML human inspired methodologies expedite the training phase, allow for smaller training sets and often improve accuracy, it does not allow for a better generalization. To cope with this, Lake et al.^15^, noted that humans can generalize successfully from a small set of examples compared to machine learning algorithms that require considerably more examples. They developed a computational model that successfully mimics these capabilities using Bayesian program learning (BPL) for learning a large class of visual concepts from just a single example.

The success of artificial intelligence in so many areas of our lives leads to the question of whether machines can be endowed with new humanoid capabilities. Such machines will need to go beyond current engineering trends in their learning techniques and capabilities; namely, they will also have to produce new knowledge, modeling, and superior generalization and adaptation capabilities^16^. A recent example of this type of machine was presented by Tenenbaum et al., who designed a machine that guides a marble through a circular maze^14^. The machine was trained using a physical engine that mimics the real system. The residual between the actual observations and the physical simulations were corrected using a statistical model (Gaussian process regression). Then, the movement of the marble in the maze was controlled using model-predictive feedback based on the combination of the physics engine and a statistical model.

This type of combination goes beyond the normal machine learning process and may be considered in human terms as machine education. According to Skinner, humans can forget facts, skills, and knowledge. Nevertheless, education enables people to recover these capabilities when facing a challenge. Skinner noted: …" education is what survives when what has been learned has been forgotten…^11^. This suggests that human education goes beyond structured learning since it provides the seeds to regrow knowledge and capabilities when called upon to cope with a problem. These seeds exist in the form of small amounts of information invariant to the problem domain.

Machine education is defined as using domain invariant physical information and models to develop a machine that solves problems obeying these physical models and using this physical information in different domains.

The educated machine described here uses a physical model of nonlinear reflectance spectra mixing and target-materials attributes to compute a training set using non-labeled data. Since the physical model, and target-materials attributes are invariant to the problem's domain, the method overcomes a fundamental problem related to supervised learning—obtaining a training set that adequately represents the problem at hand. This approach is inspired by the human education process based on acquiring a small set of tools to deal with problems from different domains. The machine's ability to acquire human characteristics, such as the flexibility to operate in different domains through generalization and abstract thinking, is studied.

## Data and methods

### Data

#### Target material identification in hyperspectral imaging

Hyperspectral imaging (HSI) is used in numerous applications, such as geophysical mapping^20–23^, cultural heritage material analysis^24^, process control^25–27^, and many others. The resulting image is a three-dimensional data cube consisting of the spatial axis (X, Y) and the spectral information (λ). Computers are vital in HSI data analysis^28–30^; target materials are identified by comparing the reflected light's spectral signature to a reference spectrum. In many cases, these algorithms are effective even when the target material only occupies a small portion of the pixel, resulting in a linear mixing of the target and background materials' spectral signatures^21^. Another possible scenario is nonlinear mixing, which occurs when the photons are subjected to multipath effects. Nonlinear mixing results in a reflectance spectrum that is a product of the background materials' spectral signature and the target material^31^. This nonlinear situation is described in the following way. Let us assume that a portion of the scene contains a single target material $\mathcalligra{m}$, and a background material $\mathcalligra{b}$. Let $R_{\mathcalligra{m}} \left( \lambda \right)$ be the reflectance spectrum of $\mathcalligra{m}$, and $R_{\mathcalligra{b}} \left( \lambda \right)$ be the reflectance spectrum of the background. This portion of the scene is sampled by pixel *i*. Let $\alpha_{i}$ be the abundance of $\mathcalligra{m}$ in this portion of the scene, $I_{{\text{i}}}^{0} \left( \lambda \right)$ is the incident radiation intensity illuminating pixel *i*, and $I_{i} \left( \lambda \right)$ is the reflected light of pixel *i*. In this case $I_{i} \left( \lambda \right)$ is the element-wise product, denoted by $\odot$, of both $\mathcalligra{m}$ and the background material $\mathcalligra{b}$:

1 $$I_{i} \left( \lambda \right) = I_{{\text{i}}}^{0} \left( \lambda \right) \odot (R_{\mathcalligra{b}} \left( \lambda \right) \odot \alpha_{i} \cdot {\text{R}}_{\mathcalligra{m}} \left( \lambda \right){ } + \left( {1 - { }\alpha_{i} } \right) \cdot R_{\mathcalligra{b}} \left( \lambda \right)$$

**Figure 1:**
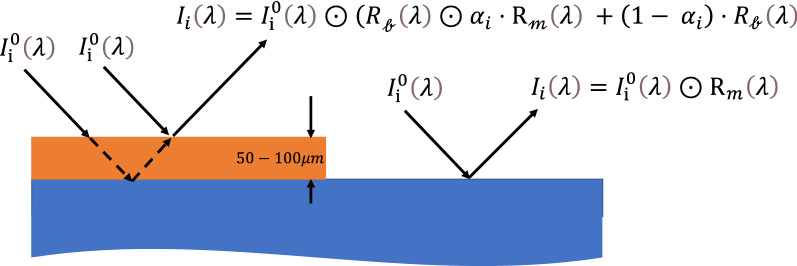
Left: An illustration of the nonlinear mixing between the reflectance spectrum from a thin (sub-millimeter) layer of the target material (red rectangle) and the spectrum arising from the background material (blue rectangle) placed behind the target material. Right: same without the target material layer.

Figure 1 illustrates this type of nonlinear mixing situation compared to a clean pixel for the simple case in which $\alpha_{i} = 1.$ Thus, Eq. (1) may be simplified to:

2 $$I_{i} \left( \lambda \right) = I_{{\text{i}}}^{0} \left( \lambda \right) \odot {\text{R}}_{\mathcalligra{b}} \odot {\text{R}}_{\mathcalligra{m}} \left( \lambda \right){ }$$

An example of the nonlinear mixing effect on the reflectance spectra of thin layers of organic materials deposited on environmental surfaces is provided in part 1 of the supplementary section. Although nonlinear mixing is a well-known phenomenon, it has received far less attention than efforts to develop unmixing algorithms for linear mixing situations^32^. Chen et al. developed a method for HSI nonlinear unmixing that implements a kernel-based learning theory. End member components at each band are mapped implicitly into the high feature space to address the photons' nonlinear interaction. Halimi et al.^33^, suggested a bilinear model combined with a hierarchical Bayesian algorithm for unmixing hyperspectral images. This model was successfully applied to real data from the Cuprite mining site (Nevada, USA) in 1997 by employing an airborne visible-infrared imaging spectrometer (AVIRIS). Dobigeon et al. compared several algorithms using computer-simulated data and real images of vegetated areas^34^. They found that the Polynomial Post Nonlinear Mixing model (PPNM)^35^, outperformed other standard models. Kendler et al. developed an algorithm capable of automatically resolving nonlinear mixing situations between a thin layer of organic material (sub-millimeter) and various background materials common in the environment^36,37^. The only input to this algorithm was ${\text{R}}_{\mathcalligra{m}} \left( \lambda \right)$, which is invariant to the measurement conditions and therefore was measured in advance in the lab and served as the reference spectrum.

Given $R_{\mathcalligra{b}} \left( \lambda \right)$, and Eq. (2), the reflectance spectrum of an unknown material *x* in pixel *i,* $R_{x, i} \left( \lambda \right)$, can be expressed as follows (3):

3 $$R_{x, i} \left( \lambda \right) = I_{i} \left( \lambda \right) \odot (I_{{0{\text{i}}}} \left( \lambda \right) \odot R_{\mathcalligra{b}} \left( \lambda \right){ })^{ - 1}$$

However, $R_{\mathcalligra{b}} \left( \lambda \right)$ is unknown and has to be estimated as accurately as possible from the scene. Furthermore, it may be impossible to assume that the illumination is uniform all over the scene. Hence, the key issue in the algorithms described in references^36,37^ is to locate a pixel *k'* in the scene without prior knowledge, which satisfies (4):

4 $$R_{{\mathcalligra{b}_{k} }} \left( \lambda \right) = R_{{\mathcalligra{b}_{i} }} \left( \lambda \right) \;and\;also I_{0,k} \left( \lambda \right) = I_{0,i} \left( \lambda \right)$$

To find *k',* three exhaustive search methods were described, resulting in a detection rate of up to 90% and a false alarm rate of less than 1% for three materials from a distance of 30 m. They also reported that the likelihood of satisfying (4) increased as the distance between pixel *i* and *k* decreased^36^.

This article presents a new, effective algorithm to resolve a nonlinear mixing situation and identify target materials. This algorithm is inspired by the human education process in which ${\text{R}}_{\mathcalligra{m}} \left( \lambda \right){ }$ are the seeds {Se} for learning, and Eqs. (1–3) are the physical model ${ }PMo$. As in the education process, {Se} and PMo are used to acquire knowledge about various scenes to generate a training set for a classifier (in this example, a Random-Forest classifier—RF)^38^, which is subsequently used to classify a new set of observations. The effects of the parameters implemented to derive the training set (education) on the classification performance are discussed.

#### Experimental setup

**Figure 2:**
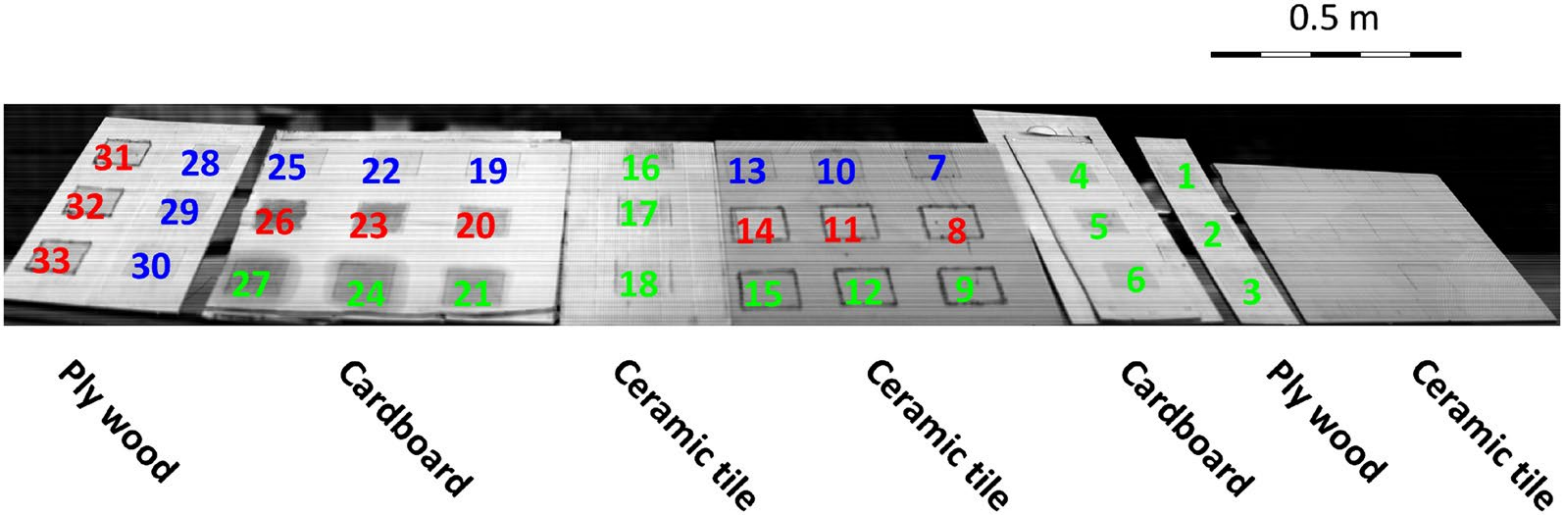
The model set of test targets. Green numbers indicate silicone oil targets; sugar targets are blue, and polystyrene targets are red. Targets 1, 4, 13, 14, 15, 25, 26, 27, 28 and 31 contained 0.5 gr of the target materials, targets 2, 5, 10, 11, 12, 22, 23, 24, 29 and 32 contained 1 gr of the target materials. The remainder of the samples contained 1.5grs of the various target materials.

The measurement was based on a set of test targets prepared in a controlled environment^36^. For the sake of completeness, a short description of the HSI and the test target containing the three model materials (sugar, silicon oil, and polystyrene) is provided. This set contained three model target materials at three concentrations on three different surfaces (ceramic tile, plywood, and cardboard). The target material spot sizes measured 10 × 10 cm and contained 0.5, 1, and 1.5 gr of the pure target material. Three materials were used: 1. sugar dissolved in hot water, resulting in a viscous (~ 50%) solution. A known volume of this solution was placed on the surface. After a few hours, the water evaporated from the surface, leaving a known amount of dry sugar film on the surface. 2. Similarly, polystyrene was deposited from a methyl ethyl ketone solution, and 3. Silicon oil (polydimethylsiloxane, PDMS) was placed directly on the surface. The resulting film thicknesses were approximately 50, 100, and 150 µm. There were 33 target spots in total, as shown in Fig. 2*.*

The reference reflectance spectra of the target materials were measured in advance using a non-imaging spectrometer (FieldSpec4TM from ASD) using a fiber-optic probe. The spectral range of the VIS-SWIR (350–2500 nm) and the sampling interval/resolution in the VIS were 1.4 nm/3 nm and 1.1 nm/10 nm in the SWIR. Since the HSI operates at 1000–2500 nm, the data in the visible range were omitted.

**Figure 3:**
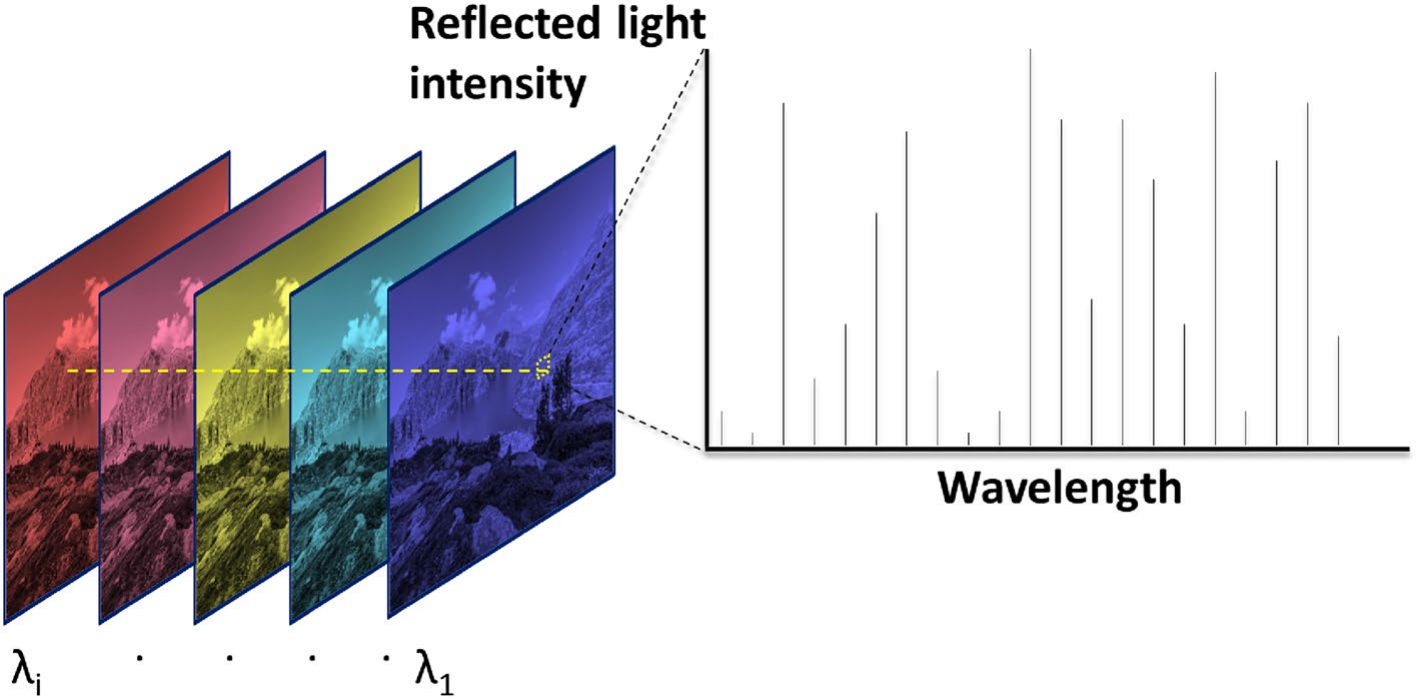
An illustration of the HSI output data. On the left are images of a scene sampled by recording the reflected light in different wavelengths resulting in a data cube. One can sample a single pixel from this data cube and obtain the spectrum of the light reflected from this pixel, as shown on the right-hand side.

HSI measurements were obtained using a line-scanner type HSI (SWIR-CL-400-N25E from Specim, Finland). The spectral range was 1000–2500 nm, and the spectral sampling interval/resolution was 5.6 nm/12 nm, using the sun as the light source. The HSI was mounted on a rotating stage and equipped with a 56 mm, F/2 lens with a 9.6° field of view. Exposure time varied from 10 to 25 ms, at a frame rate of 30 fps. Before each measurement, the HSI measured the dark current signal. Both radiometric calibration and dark current subtraction were performed automatically using the supplied control software and calibration parameters from Specim. The HSI is equipped with a Mercury-Cadmium-Telluride (MCT) focal plane array sensor with 384 by 288 pixels. Light enters the sensor through a 30 µm slit and is dispersed by the spectrometer to 288 wavelengths (channels), creating a single stripe of the scene. The data cube was constructed step by step by scanning n stripes by rotating the system n times during the measurement to create an ${\Omega } = \left| {m \times n \times l} \right|$ elements data cube. For the sensor used for this study, m = 384, l = 288, the number of samples (n), was set to be n = 2859, resulting in 1.1e6 spatial pixels. Figure 3 schematically depicts the HSI data cube. The cube was cropped down to P = 18,266 pixels and labeled according to its content. The distance between the sensor and the target was ~ 10 m. The analysis used a collection of cubes that needed to be perfectly aligned. For simplicity, the targets and the imager position were constant during the measurement of this cube collection. As the sun (the light source for these measurements) orbits the Earth, $I_{0} \left( \lambda \right)$ is not constant; additionally, the exposure time is not fixed during the measurements. As shown below, although the same set of targets and measuring device were used, these changes pose a significant challenge to a classical ML model since it lacks the ability to generalize.

### Educating the machine

Let us now define the education process using the notation above. To this end, let $\left\{ {Se} \right\}$ be a set of seeds and $PMo$, a physical model. Given a specific real-life situation with a set of real-life unclassified HSI observations $O\left( X \right)$ that behave according to *PMo*, the machine will generate a set of labeled observations using $\left\{ {Se} \right\}$ and $PMo$. Using this set of labeled observations, a classifier is computed and used to classify observations. These seeds and physical models should apply to any domain. In other words, they are general tools that enable learning for a specific mission. This process is suggested as an analogy to human education in a specific field that still enables humans to acquire tools in a changing environment.

The analysis was based on prior knowledge of the reflectance spectrum of a pure target material *x*, ${\text{R}}_{{{\text{ref}},{\text{ x}}}}$ which are the seeds for education, $\left\{ {Se} \right\}$, and several measurements of the scene—cube collection. In this work, the cube collection contained 41 cubes. Each cube contained 18,266 pixels that could contain a target material; note that at this stage, it was assumed that each pixel could only contain a single target material and a background material, whereas the entire scene could contain several materials at the same time, which for this work was three. For a given cube, {S} is the set of pixels with no target material, and {F} is the set of pixels that may contain one of the target materials. The algorithm assumes that although the cube may contain no contaminated pixels; i.e., $\left\{ F \right\}$ maybe an empty set, it always contains a set of clean pixels; thus, $\left\{ S \right\}$ is never an empty set.

For a specific data cube, a fraction, *p*, of the clean pixels were randomly selected (in this case, *p* ranged from 0.01 to 0.6). $\left\{ {Se} \right\}$ were superimposed on the $p \cdot \left| S \right|$ clean pixels using Eq. (2), resulting in a synthetic set composed of the nonlinear mixture of the target materials' spectral signatures and clean pixels containing various background materials. The set contained |S| observations for each target material and |S| observations of clean pixels. Since the clean pixels were taken from the raw data, the resulting set of labeled observations accounted for the variation in illumination intensities and other noise sources.

This set of labeled observations, obtained in a process inspired by human education, was used for the most critical ML stage, namely, obtaining a training set, in this case, for a Random Forest classifier. The random forest consisted of 12 trees since the out-of-bag classification error^37^ was low and stable for this value. The training set size and the impact of the physical model's quality on the quality of the educational process and overall performance have been studied. The remainder of the $\left[ {\left| F \right| + \left( {1 - p} \right) \cdot \left| S \right|} \right]$ pixels in the cube were classified using the computed classifier. Since the data were noisy in this case^36^, additional steps were taken to improve accuracy.

To this end, the algorithm was tuned to minimize false-positive identification; therefore, the pixel was labeled as background if the combined maximum score was lower than a predetermined threshold. This thresholding rejected some positive (true and false) identifications of the target material. This iterative training/classification accounted for the between-pixel variability and ensured that the results were not affected by a random choice of the set of {S} that was used to generate the training set. The number of iterations ranged from 1 to 30. After completing the desired number of iterations, a combined score was computed for each pixel. Several metrics to compute the combined score for the presence of target material were tested, including the arithmetic mean, median, geometric mean, and the most probable score. Using the arithmetic mean resulted in slightly improved performance; hence, it was used for this study. The effect of *p* on accuracy was also estimated. Lower *p* values reduce computation time but may not accurately represent the variability between observations, thus reducing the algorithm's ability to learn proper classification patterns.

Once all the cubes of a specific scene had been classified, each pixel's final label was determined using the most probable label across the entire cube collection. It will be shown that voting between results obtained in different conditions increases the classification reliability.

Choosing the appropriate values of *p* and the number of iterations with and without voting is an essential step in machine education since these parameters affect the quality of the training set. Subsections 2 and 3 in the supplementary material provide more details on the effects of these parameters on the overall performance.

The method described here is aimed at computing a training set; hence, it might be compatible with other classifiers that undergo supervised training. In the case presented here, the random forest model was chosen since it was found effective for datasets of reflectance spectra^39^. Additional study is required to finetune the method for different classifiers and datasets.

The main procedures of machine education are described in the following pseudocode:
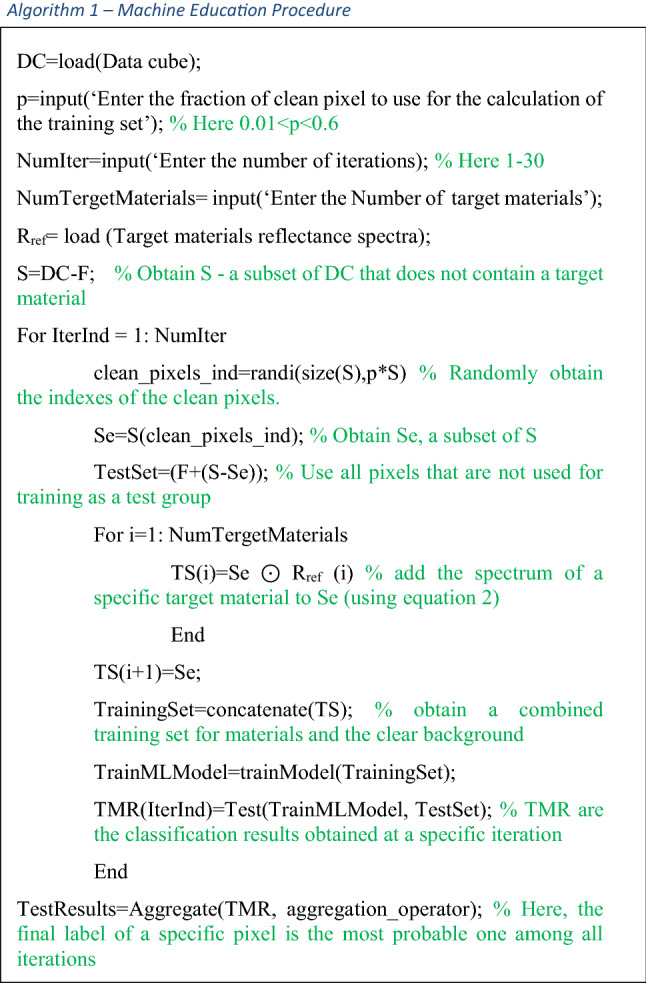


### Classifier performance evaluation

The performance of the classifier was evaluated in two stages. In the first stage, the combined score of the results of the training–testing iteration for a single cube was tuned to detect at least 90% of the targets for *p* = *0.15*, with seven iterations. The resulting combined score (0.4) was used for all other computations. In the second stage, the classification quality was evaluated for various parameters, including the number of iterations (NI), *p*, and the number of cubes used for voting. These parameters affect the learning process quality and hence are informative as to the quality of education.

Note that successful target identification was considered to have been achieved when at least one pixel in the target had been correctly identified. Similarly, even a single miss-classified pixel was considered a classification error.

A standard ML procedure was also carried out by labeling each pixel in a specific cube. Then, a training set containing 70% of the pixels was randomly chosen, and the remaining 30% were used for testing. The use of the same cube for training and testing was denoted here as 'same cube classification’ (SCC). The SCC mode of operation is an ideal case in which labeled and tested data are obtained simultaneously. The classifier was also challenged with data from other cubes, denoted, here, different cube classification' (DCC). For a cube collection with *n* cubes, there were $\frac{{\left( {n - 1} \right)n}}{2}$ possible DCC results and *n* SCC results. Hence this technique's performance (accuracy and generalizability) was evaluated using the distribution of the classification results.

## Results

**Figure 4:**
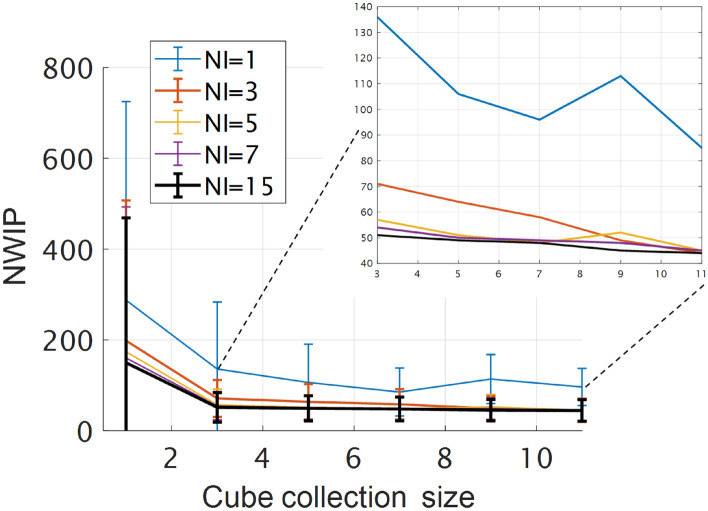
The average NWIP as a function of cube-collection size (*p* = 0.15) for various Numbers of Iterations (NI). Error bars are the standard deviation of each data point. More information on parametrization is provided in the supplementary material.

One of the educated machine's main advantages is that it does not require a labeled dataset, which is often the bottleneck in machine learning applications. This feature is beneficial for classifying data cubes from different domains, resulting from differences in illumination intensity and data acquisition parameters. Since such variations are likely to occur in outdoor measurements, an educated machine is particularly suited for such applications. Furthermore, the flexibility to move between domains makes it possible to use voting between different cubes, further enhancing classification accuracy. Figure 4 shows that voting improves accuracy. A detection probability of 97 ± 1% with 30–70 (0.2–0.4%) misclassified pixels was obtained with 7 iterations, *p* = 0.1, and 5 data cubes. Table 1 shows the classification performance for the three materials with and without applying a voting mechanism. It shows that voting on data obtained under different conditions *improves* accuracy as the number of wrongly identified pixels (NWIP) is reduced. It also points to certain differences in classification accuracy for different materials caused by variability in the signal-to-noise ratio of the reflectance spectra (see Fig. S1. in the supplementary material).

**Table 1:** The educated machine algorithm's performance (run parameters: 7 iterations, p = 0.15, voting between 5 data cubes).

Feature	Material				Voting applied?
	Sugar	Silicone	Polystyrene	Total	
Probability for detection, %	89 ± 0	98 ± 2	100 ± 0	96 ± 1	Yes
*NWIP*	13 ± 11	5 ± 3	20 ± 20	39 ± 21	
Probability for detection, %	95 ± 5	98 ± 2	100 ± 0	98 ± 2	No
*NWIP*	50 ± 78	8 ± 5	52 ± 94	110 ± 115	

Standard deviations were calculated by repeating the computations for n = 24.

**Figure 5:**
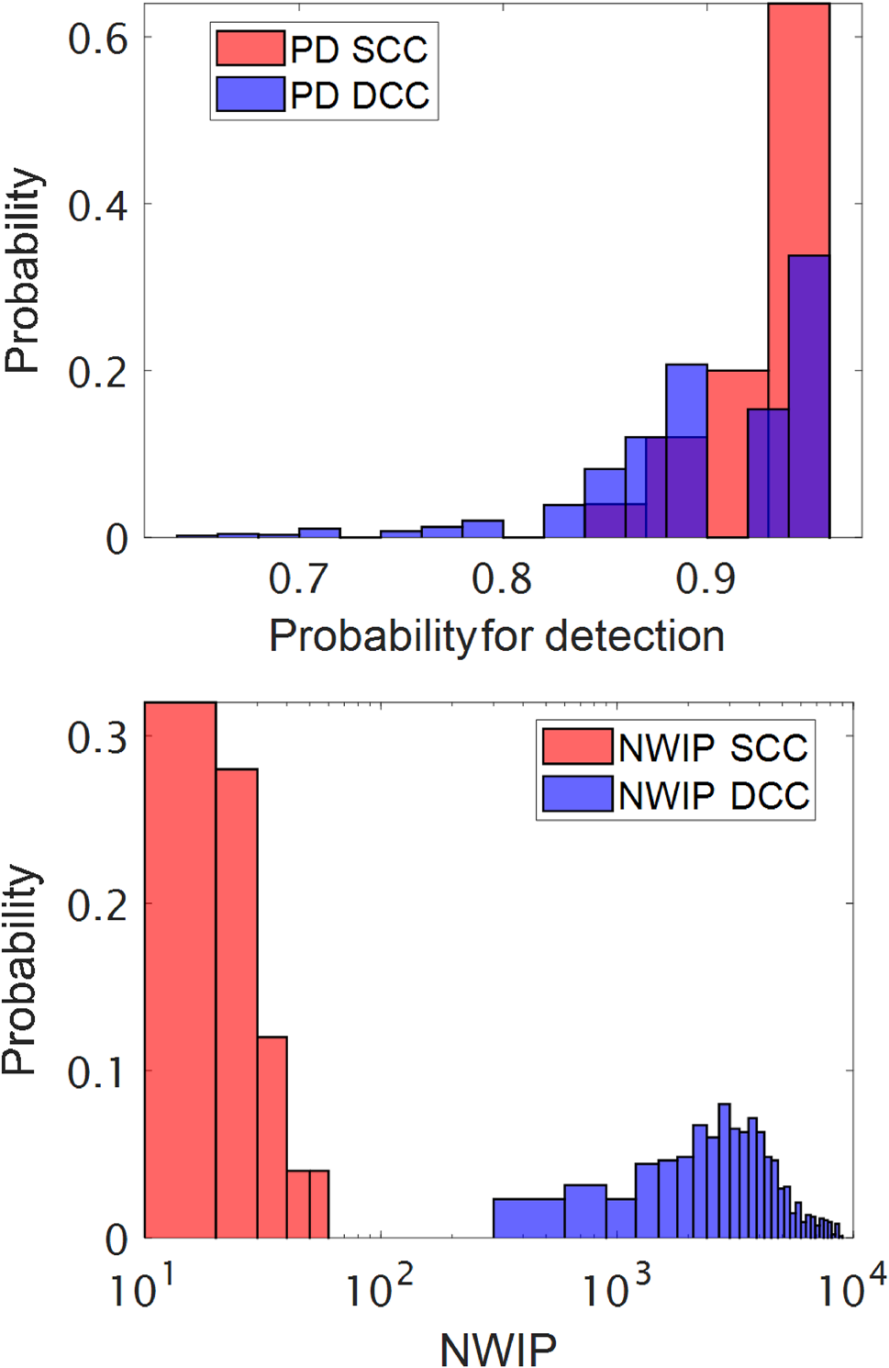
Top: target detection rate distribution using the DCC and DCC approach in conventional ML. Bottom: the same for NWIP using a logarithmic scale.

For comparison, Fig. 5 shows the classification results (NWIP distribution for SCC and DCC) obtained using conventional machine learning techniques (see “Classifier performance evaluation” section). There was a significant difference between the SCC and DCC. For the SCC, a target detection rate exceeding 90% was obtained for 84% of the data cubes. In all the other cases, the target detection probability exceeded 85%. The NWIP for the SCC approach was 10–60. The DCC approach resulted in significantly inferior results. A target detection rate above 90% was only achieved in 49% of the cases and dropped as low as 65–80% in other cases. A more dramatic effect was obtained in this case for the NWIP, which was two to three orders of magnitude higher than in the SCC approach. Table 2 compares the results obtained using the educated machine, operating at optimal parameters, and the classical machine learning using the SCC and the DCC methods.

**Table 2:** A comparison between the classical and educated machine.

Feature	Method		
	Classical SCC	Classical DCC	Educated machine
Probability for detection, %	93 ± 2	90 ± 5	96 ± 1
*NWIP*	25 ± 10	3400 ± 1800	39 ± 21

Performances are averaged for all materials; the educated machine was operated at optimal parameters.

This comparison shows that the classical machine, using the SCC approach, performs similarly to the educated machine. The probability for detection with the educated machine is slightly better than with the classical machine, and the difference in the NWIP values is within the error range. However, the conventional machine failed to generalize. This failure was evident in the DCC approach, which resulted in an average probability for detection of 90 ± 5% and an average misclassification of 3400 ± 1800. It should be noted that the SCC approach is impractical since the classical machines have to memorize the data prior to their operation; hence one has to consider the DCC as a more realistic approach. The education process created a superior machine with superior flexibility, crucial in realistic situations. This flexibility resulted from combining a nonlinear mixing physical model and the pure target materials' spectra, which is invariant to the scene's characteristics. This combination is analogous to human education, enabling adaptation to new situations without compromising performance.

## Conclusions

This work presents a new approach for increasing machines' flexibility using human-inspired training in which the machine is educated instead of being trained to memorize data. This mechanized education process is analogous to the human education process as it results in high adaptivity by obtaining relevant knowledge using a physical model and a small amount of information (seeds) invariant to the problem domain. When confronted with a new problem, the machine used these seeds and the physical model to generate the knowledge needed to create a classifier. This process was illustrated here for chemical identification using HSI utilizing the RF classification model, which is simple and effective for classifying data resulting from reflectance measurements^39^. Testing the applicability of the human-inspired method to other problems using different classifiers is part of our ongoing study.

The educated machine used a physical model for the nonlinear mixing between the uninformative background and the reference spectral signatures (seeds) to compute a classifier to detect the target materials in a realistic scenario where the signals are nonlinearly mixed. The physical model and the seeds were obtained in advance prior to the operation of the machine. Such a process is analogous to human education in that both promote the process of learning by providing tools and not by memorizing data. Educational quality was evaluated by tuning parameters such as the number of clean pixels and the number of iterations during classifier computation.

The findings presented in this manuscript suggest that machine and human education have several features in common since both improve the generalization capability due to their inherent flexibility. This capability is apparent in the realistic case in which a classical machine was trained prior to the operation—the DCC approach. In this case, the NWIP with the educated machine was two orders of magnitude lower than the classical machine. The probability for detection with the educated machine is 96% compared to 90% with the classical machine.

This capability to move between domains has long been considered the main difference between humans and machines. This work suggests that future machines can gain from education just as humans do. Hence, data scientists should focus their future efforts on educating machines rather than training them. This strategy may pave the way for a new approach to using machines in situations often considered too cumbersome for humans and too complicated for machines.

## Supplementary Information

Supplementary Information.

## Data Availability

The datasets used and/or analysed during the current study available from the corresponding author on reasonable request.
