# Supplementary material for: Hyperspectral imaging for chemicals identification: a human-inspired machine learning approach
Source: Sci Rep. 2022 Oct 20;12:17580. doi: 10.1038/s41598-022-22468-7 (PMC9584913; doi:10.1038/s41598-022-22468-7)
Supplement: Supplementary file 1 — Supplementary Information. [file 41598_2022_22468_MOESM1_ESM.pdf]

SUPPLEMENTARY MATERIAL

1. DEPICTION OF THE NONLINEAR MIXING MODEL

Fig. S 1 shows $R_x(\lambda)$ (*x* = {sugar, polystyrene, and silicone oil}) compared to as $R_{ref,\ x}(\lambda)$ obtained using equation (2) in the main text from the raw data. The similarity between $R_x(\lambda)$ obtained by unmixing, and $R_{ref,\ x}(\lambda)$ with is measured in the lab separately, indicates that this set of targets adhered to the physical model outlined in the main text.

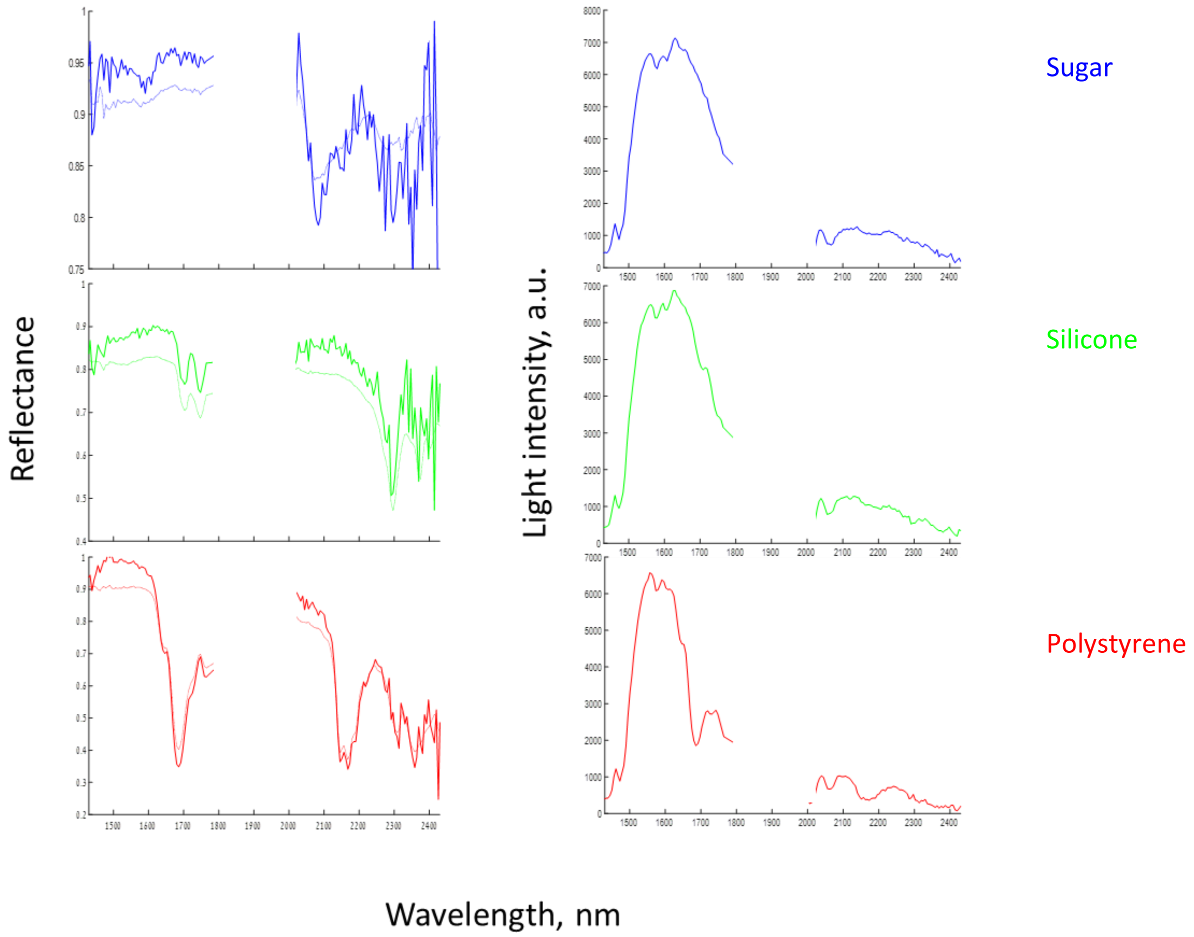

Fig. S 1. Right: the raw data as measured by the HSI, showing that the raw spectra were non-linearly mixed with the background material spectrum. The portion of the spectrum that was unusable due to atmospheric absorption is hidden. Left: $R_{ref,x}(\lambda)$ (thin dashed lines) compared to the measured spectra $R_x(\lambda)$ (solid lines) obtained from the raw data using equation (2). $R_{ref,x}(\lambda)$ was measured using a non-imaging spectrometer (FieldSpec4TM from ASD).

2. SINGLE CUBE ANALYSIS – THE EFFECT OF CLASSIFICATION PARAMETERS

The following section provides insights into the effects of education on classification accuracy before aggregating the results from several cubes through voting. Fig. S 2 shows *NWIP* as a function of *p* for various test- train iterations (NI). Each data point averages 18 different cubes; the error bars are the standard deviations of the *NWIP* values.

The *NWIP* decreased as NI, and the *p* values increased. Increasing *p* from 1 to 15% reduced *NWIP* by a factor of 2-4; a further increase of *p* had no significant effect on *NWIP*. Similarly, increasing NI from 1 to 5 reduced *NWIP* by a factor of ~2 for all *p* values; a further increase of *NI* had no significant effect on *NWIP*. The effect of *p* on the algorithm's performance can be attributed to the variability in the scene (for example, uneven illumination). The value of *NI* also affected the classification quality since the RF classifier's derivation involved a random choice of out-of-bag samples, which impacted the resulting classifier's performance. Some random choices resulted in low *NWIP* values, but others in very high values, as shown in the upper left box of Fig. S 2 for *NCy=1*. Repeating the computation, several cycles reduced this noise. Note that an NWIP value of 100 for a scene containing ~18000 pixels means that the probability of a false positive is 0.56% at a true-positive rate higher than 90%, which can be considered an accurate algorithm. The advantages of voting between data cubes are presented in the next section.

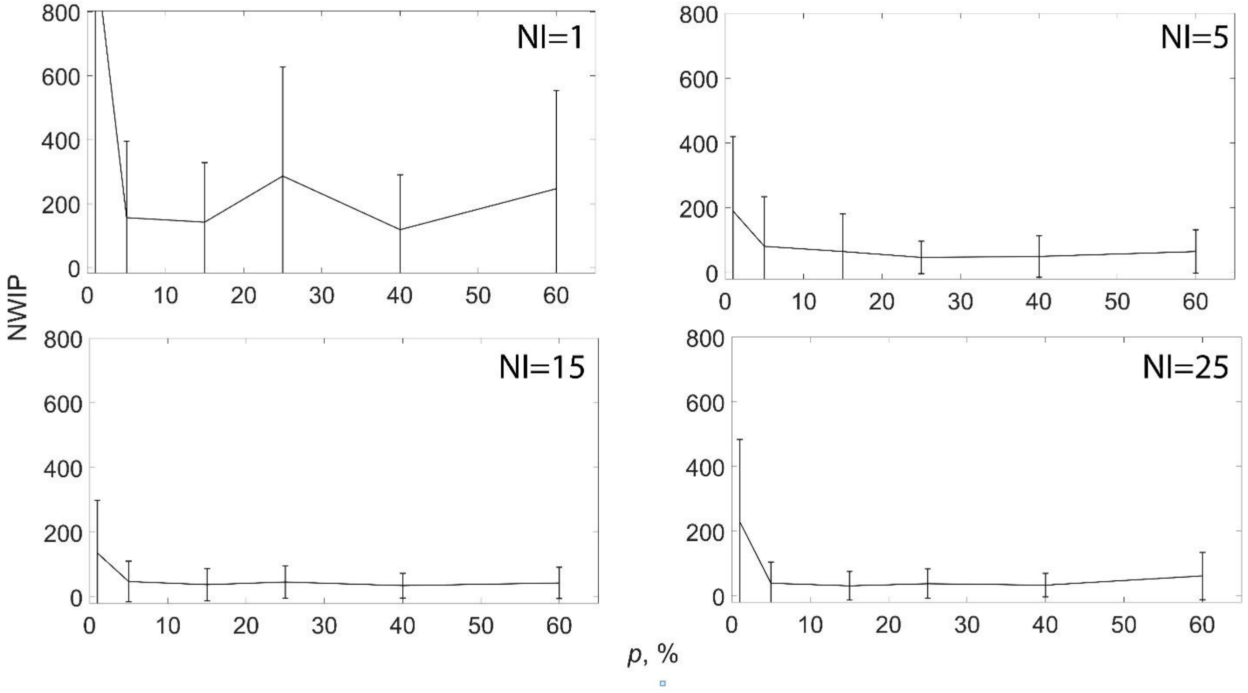

Fig. S 2: Average (n=18) NWIP as a function of Fr for various NCy values using a single data cube for analysis. Error bars are the standard deviations of the calculated NWIP values in each case.

3. *SETTING UP THE VOTING MECHANISM*

Since this method relies on seeds and a physical model that is invariant to the problem domain, the same scene can be measured at different illumination intensities and exposure conditions and use the average classification result. This section explores the effect of the parameters on accuracy in the specific case of voting between three data cubes. The main text discusses the effect of the number of cubes used for voting (cube collection size) in detail . The target detection rate and *NWIP* were calculated using several classification parameter values. Fig. S 3 shows an example of this calculation (*NI*=7) using a cube collection containing three data cubes as a function of the *p* values. Each data point in this graph represents the average of the classification results of 24 classification processes in which three cubes were

randomly selected for this analysis. The error bars are the standard deviations. The graph shows that classification reliability increased dramatically with *p* for $0.5 \le Fr \le 5\%$, whereas, above 5%, *Fr* had a negligible effect on classification reliability. At p=15% the target detection probability was 94-98% and NWIP =25-100, which is equivalent to a 0.14-0.6% false alarm rate for a cube containing 18000 pixels.

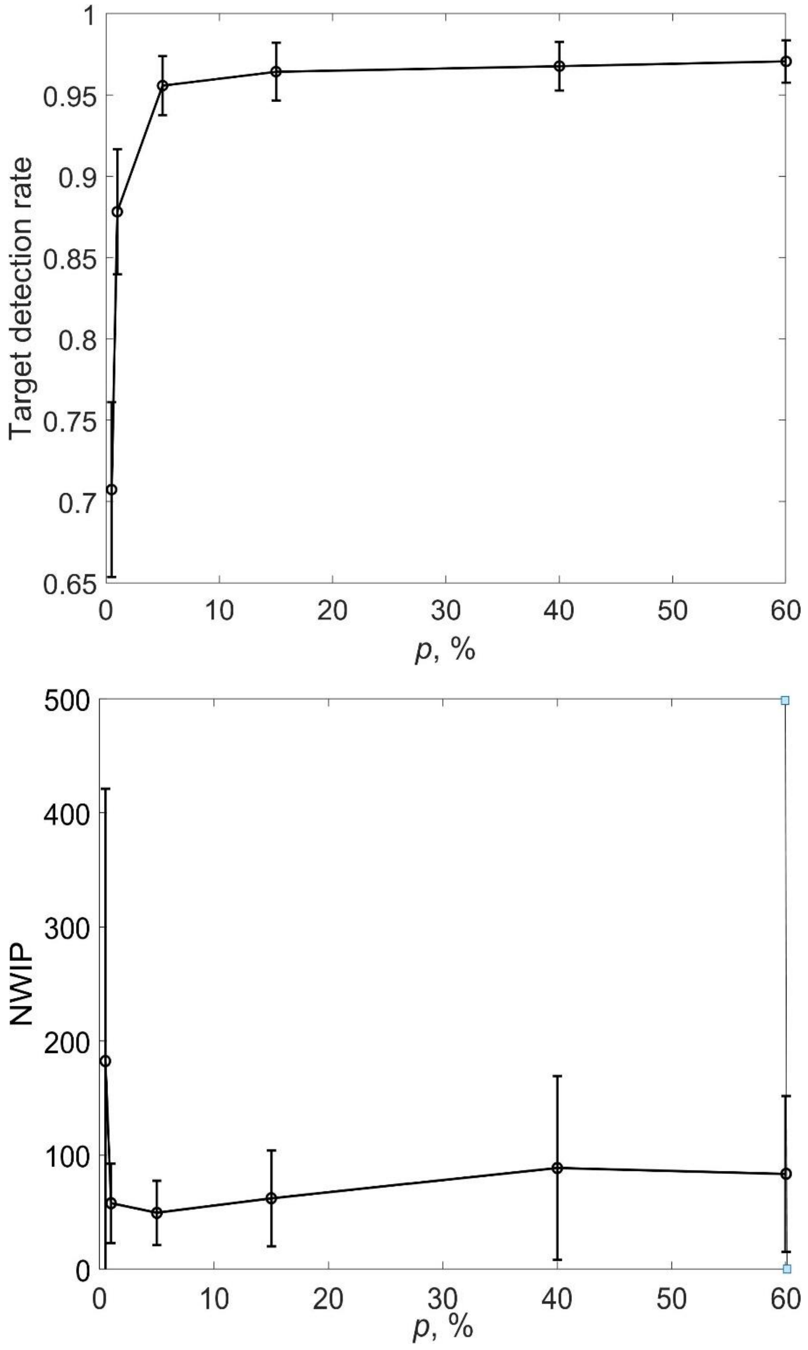

Fig. S 3: Top: average (n=24) target detection rate as a function of Fr using a cube collection comprised of 3 data cubes and seven train test iteration. Error bars are the standard deviations of the calculated detection rate: bottom, the same for NWIP.
